# Congenital Tuberculosis in a Neonate: A Diagnostic Dilemma

**Published:** 2014-10-20

**Authors:** Raj P, Sarin YK

**Affiliations:** Department of Pediatric Surgery Maulana Azad Medical College, and associated Lok Nayak Hospital, New Delhi

**Keywords:** Congenital tuberculosis, Lymphadenopathy, Ascites, Liver biopsy

## Abstract

Though tuberculosis (TB) among pregnant women is not unusual in our country, documented cases of congenital tuberculosis are rare. Diagnosis is often difficult as signs and symptoms in a neonate are non- specific. Maternal history of tuberculosis is often missed, as many of them are asymptomatic. Here we present a neonate who was operated in view of intestinal obstruction which intra operatively showed disseminated abdominal tuberculosis with infected ascites.

## CASE REPORT

A 21-day-old male neonate with birth weight of 2.65 kg was brought with complaints of non-passage of stools for 3 days and gradually increasing abdominal distention, fever and vomiting for 2 days. There was no history of delayed passage of meconium. Antenatal and perinatal period was uneventful. Child was apparently well for the first two weeks of life, but started developing refusal to feed and respiratory distress. On examination, the baby was lethargic, sick-looking, tachypneic and febrile. Abdominal examination revealed grossly distended abdomen, sluggish bowel sounds, with no guarding or rigidity. Blood investigation showed Hb 13.6g, TLC- 5300/μL, polymorphs- 63%, platelet count -69000/μL. Abdominal radiograph showed dilated bowel loops with no air fluid level or free gas. Considering Hirschsprung’s disease as a differential, barium enema was done, which was normal. The general condition of the neonate did not improve and abdominal distension worsened. Intra-operatively, there was grossly infected ascites and multiple micro-abscesses present in liver and spleen (Fig. 1) along with necrotic pus filled mesenteric lymph nodes. Biopsy was taken from liver and mesenteric lymph nodes. Ascitic fluid was sent for culture. The post-operative course was stormy; he had refractory shock to which he succumbed in few hours. Biopsy from the liver and lymph nodes received few days later revealed caseous necrosis with granuloma formation. Pus was highly sensitive for acid-fast bacilli mycobacterium tuberculosis. Mother was otherwise asymptomatic; the detailed history regarding tuberculosis could not be gained, as the parents could not be contacted.

**Figure:**
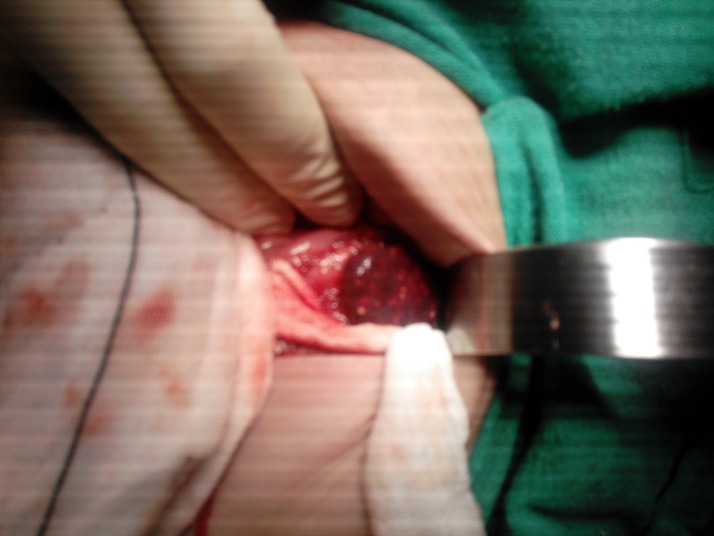
Figure 1: Multiple abscesses in spleen.

## DISCUSSION

Modes of transmission of congenital TB is thought to be acquired in three ways: transplacentally, where primary complex is in liver; aspiration of infected amniotic fluid during birth, when lungs are primary focus; and ingestion of infected material, where the primary is in the gut.[1]

Beitzke [3] in 1935, established a criteria for differentiating congenital TB from postnatally acquired TB, and it included:

1. Isolation of M. tuberculosis from the infant,
2. Demonstration of the primary complex in the liver,
3. In the absence of primary complex in the liver-
  a) Evidence of tuberculosis within days after birth.
  b) Absence of contact with a case of tuberculosis after birth.

However Beitzke criteria were developed from autopsy series and were rigid. Cantwell [1] revised these criteria in 1994, and they include:

Proven tuberculosis lesions in the infant plus one of the following:

- Lesions occurring in the first week of life,
- A primary hepatic complex,
- Maternal genital tract or placental tuberculosis,
- Exclusion of postnatal transmission by thorough investigation of contacts.

Our case meets the Cantwell’s criteria as it is a proven case of tuberculosis in a neonate with primary complex in liver.History of TB from mother couldn’t be elicited as baby presented with abdominal distention and TB was not kept in differentials. 

Signs and symptoms are usually non-specific and include respiratory distress, fever, and hepatosplenomegaly. These symptoms are often confused with other condition like sepsis and congenital infection. Our case presented with both respiratory and abdominal signs, though the respiratory distress was attributed to the gross abdominal distention. Hepato-splenomegaly though present in our case was not appreciated in view of abdominal distention. Severity of abdominal signs in our case suggests that the mode of entry was probably through the ingestion of TB infected amniotic fluid during birth process. Thereafter, dissemination occurred through the portal vein and lymphatics thus explaining the multiple liver abscess and gross ascites. Many infants with congenital TB have an abnormal chest radiograph [5], our case too had abnormality in the right lung but they were attributed to broncholitic changes and thus tuberculous origin was missed.

Congenital TB has a very high mortality rate, and those presenting before 4 weeks have mortality up to 50%.[1-4] Our case too presented within 4 weeks and expired, thus stressing on the need for high index of suspicion, early diagnosis and prompt treatment.

## Footnotes

**Source of Support:** Nil

**Conflict of Interest:** The corresponding author is an editor of the journal. This manuscript is handled independently by other editors and the author has not been involved in decision making about this manuscript.

